# Improving the accuracy of template-based predictions by mixing and matching between initial models

**DOI:** 10.1186/1472-6807-8-24

**Published:** 2008-05-05

**Authors:** Tianyun Liu, Michal Guerquin, Ram Samudrala

**Affiliations:** 1 Department of Microbiology, University of Washington, School of Medicine, Seattle, WA 98195, USA

## Abstract

**Background:**

Comparative modeling is a technique to predict the three dimensional structure of a given protein sequence based primarily on its alignment to one or more proteins with experimentally determined structures. A major bottleneck of current comparative modeling methods is the lack of methods to accurately refine a starting initial model so that it approaches the resolution of the corresponding experimental structure. We investigate the effectiveness of a graph-theoretic clique finding approach to solve this problem.

**Results:**

Our method takes into account the information presented in multiple templates/alignments at the three-dimensional level by mixing and matching regions between different initial comparative models. This method enables us to obtain an optimized conformation ensemble representing the best combination of secondary structures, resulting in the refined models of higher quality. In addition, the process of mixing and matching accumulates near-native conformations, resulting in discriminating the native-like conformation in a more effective manner. In the seventh Critical Assessment of Structure Prediction (CASP7) experiment, the refined models produced are more accurate than the starting initial models.

**Conclusion:**

This novel approach can be applied without any manual intervention to improve the quality of comparative predictions where multiple template/alignment combinations are available for modeling, producing conformational models of higher quality than the starting initial predictions.

## Background

Comparative modeling methods are based on the observation that proteins related by evolution generally share similar three dimensional (3D) structures [1,2]. Therefore, the 3D models of a protein without an experimentally determined structure (target) can be built using alignments of the target sequence to one or more proteins with experimentally determined structures (templates). Currently, it is the most accurate approach for protein structure prediction, although there are significant bottlenecks that need to be overcome before models comparable to experimental results can be produced generally [3-5]. First, the accuracy of comparative predictions depends on the quality of the sequence alignments between the target and the templates sequences [1,2]. The results from the sixth Critical Assessment of Structure Prediction (CASP6) experiment indicates that even though there has been substantial progress in the quality of alignments, it has not resulted in an obvious improvement in the quality of the final model [6,7]. Second, even if the best template structures can be identified and used, at low sequence identities, the resulting models are still quite distant from the corresponding experimental structure. This highlights the importance of the need to develop methods for refining comparative predictions derived from those templates.

For a given target protein, several different template structures are usually available. The sensitivity of template identification and the accuracy of an alignment are enhanced when using sequence profiles constructed from multiple templates/alignments, though in some cases strong sequence signals for an accurate individual template may end up being ignored [8]. Some modeling programs incorporate information from multiple templates: For example, the MODELLER program builds models by satisfaction of spatial restraints from several initial models constructed using multiple templates/alignment combinations [9]. However, a given template/alignment combination is unique in its similarity to the target protein in different ways [10], and thus comparative predictions using different templates/alignments produce different structural models for a given target protein. Even when using the same template/alignment, different modeling methods can yield different models due to variations in the side chain and loop building processes [11].

Usually, an initial comparative model derived from a single template/alignment rarely has all the information necessary for further structural and functional analysis. Alternative models derived from other templates/alignments may provide additional key structural and functional information, even if the global structural similarity is not significant [4]. Thus even if one possessed an ideal discriminatory function to select an initial model most resembling the experimental structure, such a model may be limited in use for understanding of the function of the protein. We therefore ask the question: Given a set of models derived from multiple templates/alignments for a target protein, how can one take into account all of the information in a rational way to produce more accurate models?

The methods for templates/alignments recombination have been extensively applied to template-based modeling [12-16]. 3D-SHOTGUN was one of the first fully automated methods designed to assemble hybrid models by using the recurrent structural information from initial models generated using different fold recognition methods [13]. The rationale of 3D-SHOTGUN is that recurring structural features observed in independent initial models are more likely to represent the experimental structure of a protein. The *In Silico *Protein Recombination method developed by Bates et al. employs a genetic algorithm to recombine initial models with crossover points outside the regions of secondary structure, and mutation by averaging the coordinates of two initial models [14]. The FRANKENSTEIN'S MONSTER is also an approach assembling fragments derived from comparative modeling and fold recognition [15]. The novelty of this approach is that the hybrid models are used for a further step of local realignment of uncertain regions [15,16]. These methods proved to be effective at exploiting the recombination of multiple templates/alignments. However, the quality of the initial models is the upper limit for the quality of the final model.

We previously developed a graph-theoretic clique finding (CF) approach to handle the large conformational space of main chain and side chain possibilities resulting from the interconnected nature of interactions in protein structures [17]. The approach has worked well in blind prediction comparative modeling experiments for constructing variable main chains and side chains [18-20]. The major difference between CF and other methods for templates/alignments recombination lies in the graph-theoretic representation, which considers the recombination of fragments systematically, avoiding the need for following a trajectory through the rough energy landscape. Unlike other methods which evaluate hybrid models by statistical potentials directly, the CF method incorporates pre-calculations of the fitness of each interaction of main chains and side chains. Thus the computational cost of evaluation is reduced, allowing more combinations to be considered.

Here, we employ the CF approach in a fully automated manner to mix and match segments of different initial models for a given target protein. These initial comparative models may be obtained from different templates/alignments or different comparative modeling methods. We found that using the CF approach of mixing and matching initial models contributes to the improved accuracy of comparative predictions at the recent CASP7 experiments relative to the initial models used.

## Methods

The objective of our fully-automated approach is to find the optimal set of interactions in a protein structure that can be obtained by mixing and matching a set of comparative models. Optimal combinations of possible main chain and side chain conformations were explored and selected using a graph-theoretic clique finding (CF) approach and a residue-specific all-atom discriminatory function (RAPDF) [17,21].

### A graph-theoretic clique finding (CF) approach for exploring protein conformational space

In this approach, each possible conformation of a residue represents a node in a graph; edges are then drawn between nodes (representing pairs of possible residue conformations) that are consistent with each other. This is accomplished by following three rules: (1) Packing consistency is maintained by not drawing edges between nodes with atoms that clash. (2) Main chain consistency is maintained by splitting the complete main chain conformation into segments, with each segment having one or more possible conformations. If two nodes representing conformations of a residue are within the same main chain segment, then an edge is drawn between the nodes. An edge is drawn between nodes from different main chain segments if they are close to each other in 3D space. (3) An edge is not drawn between different possible side chain conformations of the same residue.

Each node is given a weight based on the strength of the interaction between its local main chain and side chain atoms, and each edge is weighted based on the strength of the interactions between the atoms of the two nodes. The interaction strength is calculated using the all-atom discriminatory function RAPDF [17,21]. Once the entire graph representing the various main chains and side chains is constructed, all the maximal sets of completely connected nodes (cliques) are found using the Bron & Kerbosch clique finding algorithm [22]. The cliques with the best weights represent the optimal combinations of the various main chain and side chain possibilities. A complete detailed description of the method is given in [17].

### Selection of evaluated targets and construction of initial comparative models

Targets from the CASP7 experiment were used to evaluate the effectiveness of our protocol. A total of 40 test targets were selected using two criteria: (1) Two or more templates with an identity of at least 20% were available; (2) Five initial models share a reasonable similarity between each other, with their Cα root mean square deviation (CαRMSD) between each other better than 10 Å.

For each target, sequence alignments were obtained from the Bioinfo 3DJury server [23]. For each alignment, one initial comparative model was generated by using programs in the RAMP software suite [18-20]. To build conformations for the structurally conserved regions, residues that were identical in the target and the template proteins were generated by copying atomic coordinates for the main chains and the side chains; residues that differed in side chain type were constructed by using a minimum perturbation technique. To build conformations for the structurally variable regions, the programs mcgen_exhaustive_loop and mcgen_semfold_loop from the RAMP suite were used. The former generates conformations by exhaustively enumerating all possible main chain conformations using a 14-state ϕ/ψ model and selecting the best ones using the RAPDF discriminatory function [21]. The latter uses a fragment replacement using a Monte Carlo with simulated annealing procedure to find the best combinations of these fragments [18-20]. Alternate side chain conformations for each residue in the complete conformation were generated using the SCWRL3 software [24].

Additional initial models were also obtained from the CAFASP5 experiment [25] after examining the alignments to obtain extra variability in templates/alignments and prediction methods to ensure all residues had at least one possible conformation. To avoid side chain conflicts in the process of mixing and matching, some side chain possibilities were optimized using the SCWRL3 software.

### Defining crossover points for mixing and matching

Using the CF method for comparative modeling leads to a natural definition for crossover points [17]. Crossover points are those positions where mixing and matching between different main chain segments could occur in a self-consistent manner, without causing gross clashes or distortions of the protein structure (typically across secondary structure elements). In the case of mixing and matching between different initial models, the crossover points were defined based on multiple structural superpositions. In our original publication [17], the crossover points were determined by stretches of the main chain where the distance between the equivalent Cα atoms (Cα distance) was less than 1.0 Å. Exceptions to the 1.0 Å limit were handled in a subjective manner by visual inspection of the superpositioned structures.

**Figure 1:**
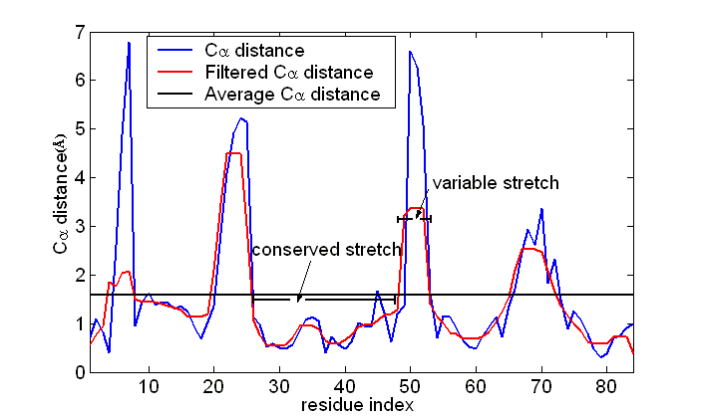
**Determination of crossover points**. The Cα distances between different initial models (blue) are transformed into an envelope of these Cα distance variations (red) using the median filter formula [26]. The intersections of the envelope and the threshold (black) that is the mean of all these Cα distances is used as crossover points for mixing and matching between different initial models. Conserved stretches are those with a lower average Cα distance, and variable stretches are those with a higher average Cα distance [see the Method section on mixing and matching between two initial models].

In this study, we innovated upon the method by implementing a step for automated crossover identification. The main chain was first divided into segments according to the spatial proximity of each equivalent Cα atoms between different initial models. The median filter [26] considered each Cα distance in the sequence in turn and looked at its nearby neighbors to decide whether or not it was representative of its surroundings. The value of each Cα distance was then replaced with the median of its neighboring values, instead of the mean of those values. Thus the unrepresentative Cα distances in a neighborhood did not affect the median value significantly. Using the median filter formula, the Cα distances between different initial models were transformed into an envelope of these Cα distances variations (Figure 1). The intersections of the envelope and the threshold that was the mean of all these Cα distances were then used as the crossover points for mixing and matching between different initial models. For multiple initial models, envelopes derived from every two models were averaged and used for deciding the crossover points.

### Mixing and matching between two initial models

From a set of initial models for each target, the two best models were selected using RAPDF. For these two initial models, multiple sets of crossover points were defined. In families of homologous structures, there are usually regions of the main chain that are very similar to each other and regions that are structurally variable, representing evolutionary insertions and deletions. Given a set of the crossover points, each initial model could be considered as an ensemble of stretches representing structurally similar or variable regions. The average Cα distances of the corresponding elements from the two initial models were calculated. Conserved stretches are those with a lower average Cα distance, and variable stretches are those with a higher average Cα distance (Figure 1).

Variable stretches may result either from different templates or different alignments used for the modeling of a particular region [27-29]. They may also result from different modeling procedures for the structurally variable regions [28,29]. For each variable stretch, an extra set of possible conformations were generated for the main chain regions. This was accomplished using the mcgen_semfold_loop program in the RAMP suite. Possible alternative conformations of the variable stretches were included in the list of conformations for mixing and matching.

Given each set of crossover points and its corresponding list of conformations, which includes the two initial models and the possible conformations for the variable stretches, the CF approach was used to obtain an optimized mosaic model. For each target, several "CF models" were generated and refined using the ENCAD software [30,31]. The best conformation was selected from among these CF models using RAPDF.

### Mixing and matching between multiple initial models

In theory, the above approach can be generalized to an arbitrary number of templates if all the initial models generated using them have similar crossover point locations. In practice, this is not the case since some members of a particular protein family are quite similar to each other and others are quite distant.

To develop a generalized procedure for mixing and matching between several initial models, we first superimposed all the initial models and calculated all-against-all CαRMSDs. To allow mixing and matching between any two models, a certain level of structural similarity between them is required. In the pool of all initial models, those with CαRMSDs less than 2 Å, 4 Å, 6 Å and 8 Å to each other were grouped together respectively for mixing and matching. Given a group of initial models, the crossover points were first determined as before and the CF approach was used to generate refined models that represented the optimal combinations of the initial models. All the CF models were then energy minimized using ENCAD. The best scoring CF model selected by RAPDF was considered the most native-like one.

### Evaluation of prediction accuracy

CF models were submitted to the manual and automated CASP7 and this study analyzed only these models. Other methods were also used for our CASP7 submissions, but in this work we analyze models submitted using the CF method. The experimental structures for all CASP7 targets have been made available. For all the initial models and the refined CF models of each target protein, their CαRMSDs to the corresponding experimental structures were calculated for evaluating the accuracy of predictions. The Wilcoxon sign rank test [32] was conducted to detect the significance of the differences between the qualities of initial models and those of the CF models of each target protein. This nonparametric test makes no assumptions about the parameters of the population distributions from which data are drawn. We hypothesized that the accuracies of the CF models are lower than or the same as those of the initial models. The calculated *P*-value from the Wilcoxon sign rank test was used to accept or reject this hypothesis.

## Results and discussion

### Mixing and matching between two best initial models

We first assessed the effectiveness of the CF method for mixing and matching between two initial models. In the mixing and matching process, several CF models were generated as a result of multiple sets of crossover points. The best scoring CF model selected by the discriminatory function RAPDF is referred to as "CF-R"; and the best initial model selected by RAPDF is referred to as "IN-R".

**Figure 2:**
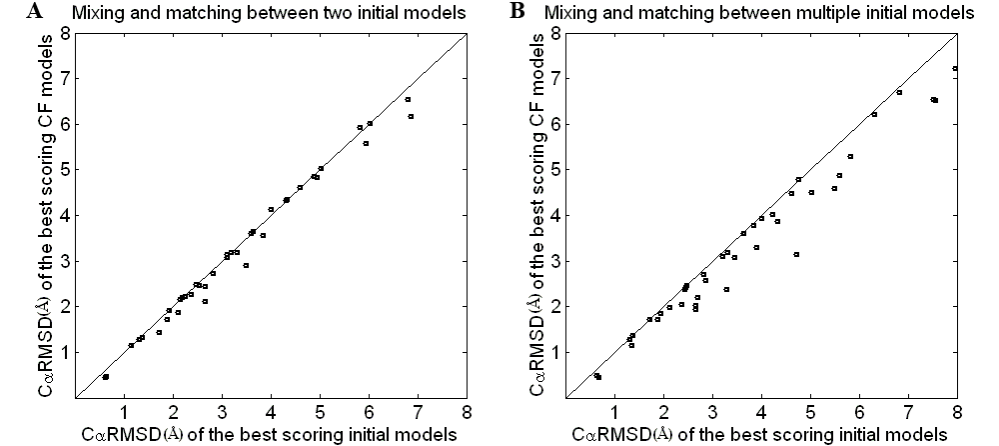
**Evaluation of the effectiveness of mixing and matching initial models**. The accuracies of the initial comparative predictions and the refined CF models are evaluated with the CαRMSD measurements of models for 40 CASP7 targets [see Additional file 1: Table 1]. For each target, the CαRMSD of the best scoring CF model is compared and plotted against that of the best scoring initial model. The accuracies of the best scoring CF models are consistently higher than or equal to those of the best scoring initial models. Mixing and matching between two initial models (A) leads to an average improvement of 0.2 Å CαRMSD between the best scoring CF models and the best scoring initial models. Mixing and matching between multiple models (B) leads to an average improvement of 0.4 Å CαRMSD. The targets with the best improvements for the two methods are 0.7 Å and 1.6 Å CαRMSD, respectively.

In terms of the CαRMSD to the experimental structure, the accuracies of CF-R are slightly but consistently higher than those of IN-R for 36 of the 40 targets (Figure 2A). The most significant improvement between the accuracies of CF-R and IN-R is 0.7 Å. When CF-R and IN-R for the 40 targets are considered as two pools of samples, the average improvement between the accuracies of CF-R and IN-R is about 0.2 Å and the *P*-value between CF-R and IN-R is 1.1*10^-5^. Thus CF models are more accurate compared to initial models at a significance level of 0.01.

### Mixing and matching between multiple initial models

We then evaluated the ability of our method to handle multiple initial models simultaneously. For each target protein, several CF models were generated where each CF model consisted of an ensemble of segments originating from two to five different initial models. The best initial models and the best CF models were selected by RAPDF for each target. In terms of the CαRMSD to the experimental structure, the accuracies of CF-R are always higher than or equal to those of IN-R (Figure 2B). The most substantial improvement is 1.6 Å. This suggests that selecting multiple initial models may introduce optimized template/alignment combinations to the process of mixing and matching. When CF-R and IN-R for the 40 targets are considered as two pools of samples, the average improvement between CF-R and IN-R is about 0.4 Å and the *P*-value between CF-R and IN-R is 7.3*10^-12^. Thus the CF models are more accurate compared to the initial models at a significance level of 0.01.

Because of the limitations of current discriminatory functions, most comparative modeling methods cannot always recognize the most native-like conformations as the best models from initial comparative predictions. We therefore asked the question: what is the efficacy of selecting the most native-like conformation from a pool of CF models generated from the mixing and matching process?

We conducted further analysis on all available CF models generated through the mixing and matching process, and all initial models from which the CF models were derived. Our tests showed that the odds of selecting the most native-like model from initial models are 20–30%, while those from CF models are 60–70% [see Additional file 1: Table 1]. Our results indicate that the mixing and matching between initial models improves the distributions of near-native conformations. Comparison between the best available initial models and the best refined CF models indicates an improvement of only 0.1 Å [see Additional file 1: Table 1]. However, because of the accumulation of native-like conformations during the process of mixing and matching, the discriminatory function discriminates the most native-like ones (best models) in a more effective manner (Figure 2). That is, the mixing and matching process yields final conformations of higher quality than the initial predictions, indicating the effectiveness of the method. In this study, RAPDF was the only the discriminatory function used in the procedure. For future work, inclusion of other discriminatory functions may improve the effectiveness of the mixing and matching process.

### The advantages of mixing and matching between templates/alignments

In previous studies, our graph-theoretic method has been fairly successful at handling the interconnectedness problem to build non-conserved main chains and side chains [13,14]. This study investigates the method's usefulness in handling multiple templates/alignments. Using multiple template/alignment combinations is often useful in comparative modeling. However, if a region of the alignment is incorrect but is assumed to be correct, then further model building cannot fix the error. Furthermore, it is difficult to choose which template/alignment combination to use for which regions in a preliminary prediction. In this regard, the CF method evaluates all possible combinations of the various templates/alignments, by taking into account the interconnectedness of the 3D protein structures. Thus it has the potential to select the correct template/alignment and find the best conformation for each substructure, resulting in an optimized conformational combination of substructures.

### Optimized conformation ensemble representing the combination of best predicted secondary structures

**Figure 3:**
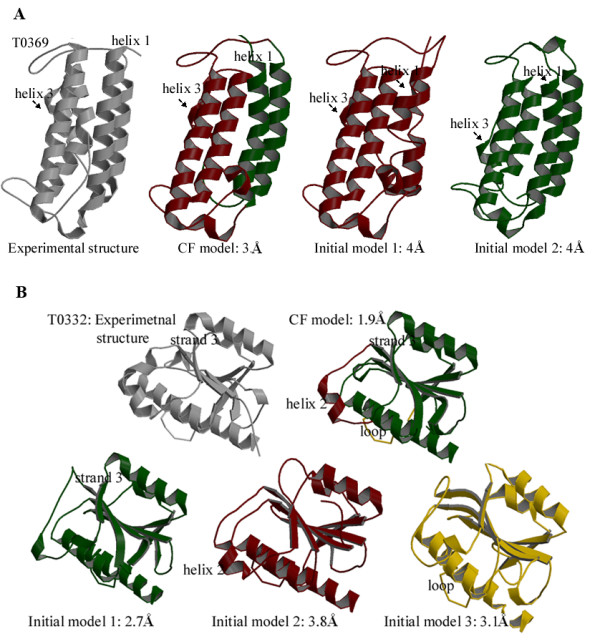
**Examples illustrating optimal mixing and matching of the best predicted secondary structures**. **A**. Comparative models of CASP7 target T0369 (PDB identifier 2H4O) are shown to explain the process of mixing and matching between two initial models. The backbone ribbon representations of the experimental structure and the two initial models are indicated as grey, green and red, respectively. Helix 1 of initial model 1 (red) is not accurately modeled, but helix 3 is predicted reasonably, resembling the experimental conformation most. Helix 1 of initial model 2 (green) is properly modeled, but helix 3 is not predicted in correct conformation. The CF method incorporates helix 3 from initial model 1 and helix 1 from initial model 2, resulting in a CF model that is improved by 0.4 Å CαRMSD relative to the experimental structure. (Figures were prepared with Molscript [34] and Raster3D [35].). **B**. Comparative models of CASP7 target T0332 (PDB identifier 2HA8) are shown to explain the process of mixing and matching between multiple initial models. The backbone ribbon representations of the experimental structure (grey) is shown compared to the refined CF model. The CF method assembles β-strand 3 from initial model 1. (green), helix 2 from initial model 2 (red) and a major loop at the bottom of the central β-sheet from initial model 3 (yellow), which are the best predicted substructures in the three initial models, respectively. This produces a CF model that is improved by 0.8 Å CαRMSD relative to the experimental structure.

Figure 3 illustrates how mixing and matching between different initial models improves the accuracies of comparative predictions. Figure 3A shows comparative models of target T0369 to explain the process of mixing and matching between two initial models. The experimental structure of T0369 consists of five major α-helices, with a kink in helix 2. Helix 3 is the shortest one, but it is misaligned in most of the initial predictions. In initial model 1 (CαRMSD of 4.6 Å), helix 1 is not accurately modeled, but helix 3 is predicted reasonably, resembling the experimental conformation most. In initial model 2 (CαRMSD of 4.3 Å), helix 1 is properly modeled but helix 3 is not predicted in correct conformation. The CF model takes fragments of helix 3 from initial model 1 and helix 1 from initial model 2, resulting in an ensemble of fragments representing the best predicted secondary structures, producing a CF model that is improved by 0.4 Å CαRMSD to the best initial model.

The results for another CASP7 target T0332 (Figure 3B) illustrates the process of mixing and matching between multiple models. In the experimental structure for T0332, the central β-sheet is flanked on both sides by a total of six α-helices. Figure 3B shows that CF model takes into account the information from the three initial models, assembling its β-strand 3 from initial model 1, helix 2 from initial model 2 and a major loop at the bottom of the central β-sheet (highlighted in yellow in Figure 3B) from initial model 3, which are the best predicted substructures in the three initial models, respectively. The CαRMSDs of the three initial models to the experimental structure are 2.7 Å, 3.1 Å and 3.8 Å; while the CαRMSD of the CF model is 1.9 Å, with an improvement of 0.8 Å.

Figure 3 indicates that the mixing and matching process finds the best secondary structures for each substructure and optimizes the interactions between them. That is, this method improves the quality of comparative predictions by constructing a conformational ensemble of the best secondary structures for each substructure.

### Optimized conformation for structurally variable regions

Reliably building the structurally variable regions remains a formidable problem in comparative modeling [1,2]. Structurally variable regions cannot be aligned to the template sequences because of insertions and deletions, and cannot be modeled by using the template structures. Thus these regions will inevitably be built with lower accuracies than the rest of the structure. In our method, a set of alternative conformations were generated for each variable region. Together with the original conformations of the initial models, these alternative conformations for the structurally variable regions were used in the process of mixing and matching. The selection of a conformation for the corresponding segments by the CF method was made on the basis of the best scoring cliques, which makes use of knowledge of the correct environment for the surrounding structure.

**Figure 4:**
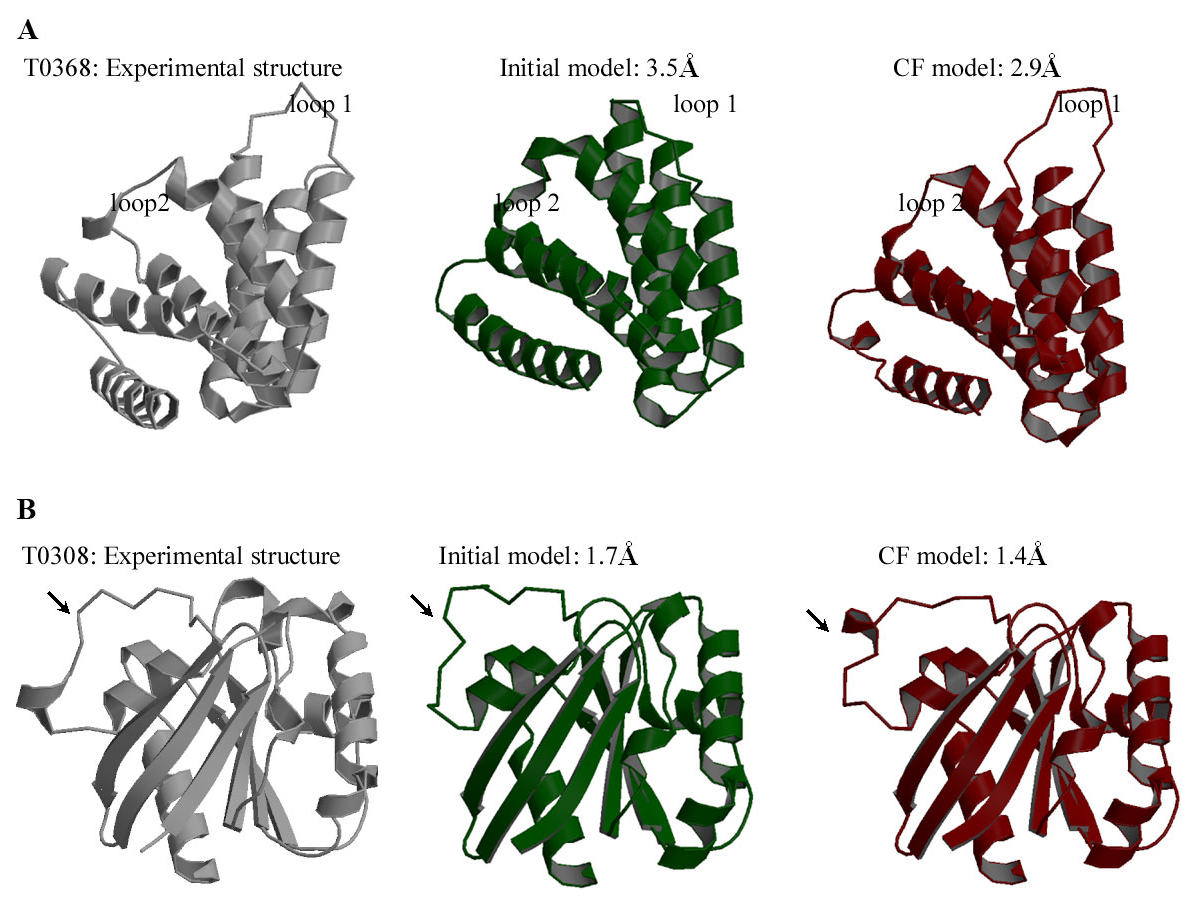
**Examples illustrating refined structurally variable regions**. **A**. Shown are the backbone ribbon representations of the experimental structure (grey) of CASP7 target T0368 (PDB identifier 2HR2), the refined CF model (red), and the initial comparative prediction (green). The two structurally variable regions (loop 1 and loop 2) of the CF model (red) show more native-like conformations compared to those of the initial model (green), which results in an improvement of 0.6 Å CαRMSD for the CF model. **B**. Shown are the backbone ribbon representations of the experimental structure (grey) of CASP7 target T0308 (PDB identifier 2H57), the refined CF model (red), and the initial comparative prediction (green). The structurally variable region (indicated by an arrow) of the CF model (red) shows a more native-like conformation compared to that of the initial model (green), which results in an improvement of 0.3 Å CαRMSD for the CF model.

Figure 4 shows how mixing and matching improves the quality of predictions for the structurally variable regions. The experimental structure of CASP7 target T0368 (Figure 4A) constrains a long loop rotated away from the core structure (loop 1). The conformation of this segment is predicted accurately in none of the initial models, whereas the CF model includes an alternative conformation that was rebuilt taking into account information from the initial models. The other structurally variable region (loop 2) in the CF model also shows a more accurate conformation after being rebuilt by the CF method. These rebuilt segments represent the native-like conformation more and thus the CF model is improved by 0.6 Å. In the other example, the CF model of T0308 (Figure 3B) shows an improvement of 0.3 Å by choosing an alternative conformation for the structurally variable region. These example results (Figure 4) represent a substantial improvement in building structurally variable regions.

Our results indicate that the CF method finds the most reasonable conformation for the structurally variable regions, thereby improving the quality of comparative predictions. The advantage of the CF method is that it evaluates multiple conformations of structurally variable regions together with multiple options in its environment simultaneously, thus allowing for some of the context sensitivity that determines interconnected protein conformation changes.

## Conclusion

In this study, the CF method is applied without any manual intervention, thus it should be effective to improve the quality of comparative predictions where multiple template/alignment combinations are available for modeling. It is available at [33]. Our extensive benchmarking on the 40 proteins shows that this fully automated process improves the accuracy of predictions through mixing and matching between two or more initial models. The average improvement between the refined CF models and the corresponding initial comparative predictions is about 0.4 Å. Contributions and prospects to the improvement include: (1) The automated method evaluates all possible combinations of available templates/alignments at the 3D level; (2) The automated stretch-finding program allows exploring all possible crossover options; (3) The process improves the distributions of near-native conformations; (4) The CF-method finds the best secondary structures for each substructure and optimizes the interactions between them; (5) The CF method searches the most reasonable conformation for the structurally variable region by evaluating multiple conformations in a context-sensitive manner. Overall our automated method produces refined models of higher quality than the starting initial predictions.

## Authors' contributions

TL carried out the computational experiments and drafted the manuscript. MG helped develop the web server. RS developed the idea and provided intellectual guidance and mentorship.

## Supplementary Material

Additional file 1 **Supplementary Material**. Tables 1 & 2. Click here for file
