# Supplementary material for: Improving the accuracy of template-based predictions by mixing and matching between initial models
Source: BMC Struct Biol. 2008 May 5;8:24. doi: 10.1186/1472-6807-8-24 (PMC2424052; doi:10.1186/1472-6807-8-24)
Supplement: Additional file 1 — Supplementary Material. Tables 1 & 2. [file 1472-6807-8-24-S1.pdf]

Additional file 1

Table 1. Summary of the initial models and the refined CF models for 40 CASP7 targets.

The accuracies are evaluated using the CαRMSD (Å) between the model and the corresponding experimental structures. The best scoring CF model selected by the discriminatory function RAPDF is referred to "CF-R"; and the best initial model selected by RAPDF is referred to as "IN-R". For mixing and matching between multiple initial models, the average improvement between CF-R and IN-R is about 0.4 Å and the *P*-value from the Wilcoxon sign rank test between CF-R and IN-R is $7.3*10^{-12}$. Significant improvements between CF-R and IN-R (>= 0.1 Å) are observed in 28 of 40 proteins (in bold font). The most substantial improvement is 1.6 Å. For mixing and matching between two initial models, the average improvement between the accuracies of CF-R and IN-R is about 0.2 Å and the *P*-value between CF-R and IN-R is $1.1*10^{-5}$. Significant improvements between CF-R and In-R (>= 0.1 Å) are observed in 16 of 40 proteins (in bold font). The most substantial improvement is 0.7 Å.

Target	Mixing and matching between multiple initial models				Mixing and matching between two initial models			
	Best initial model	Best CF model	Best scoring initial model	Best scoring CF model	Best initial model	Best CF model	Best scoring initial model	Best scoring CF model
T0346	0.52	0.45	**0.68**	**0.45**	0.52	0.45	**0.63**	**0.45**
T0290	0.63	0.47	**0.65**	**0.50**	0.63	0.47	**0.65**	**0.48**
T0345	1.16	1.16	**1.36**	**1.16**	1.16	1.16	1.16	1.16
T0315	1.31	1.29	1.31	1.29	1.28	1.27	1.31	1.29
T0305	1.37	1.37	1.37	1.38	1.37	1.33	1.37	1.33
T0302	1.6	1.62	**1.88**	**1.71**	1.60	1.66	**1.88**	**1.72**
T0308	1.66	1.71	1.72	1.72	1.72	1.43	**1.72**	**1.44**
T0291	1.74	1.61	**3.32**	**3.19**	1.74	1.61	**3.32**	**3.19**
T0324	1.92	1.92	**2.38**	**2.04**	1.92	1.90	1.92	1.92
T0328	1.94	1.85	1.94	1.85	1.94	1.86	**2.11**	**1.87**
T0340	1.94	2.00	**2.67**	**2.03**	1.94	2.11	**2.67**	**2.12**
T0288	2.12	1.99	**2.13**	**1.99**	2.16	2.16	2.16	2.16
T0303	2.20	2.21	**2.71**	**2.21**	2.20	2.20	2.20	2.20
T0295	2.37	2.43	2.46	2.43	2.45	2.46	2.54	2.47
T0359	2.37	2.37	2.43	2.37	2.37	2.27	**2.37**	**2.27**
T0362	2.40	2.01	**3.29**	**2.38**	3.11	3.07	3.11	3.09
T0335	2.45	2.44	**3.46**	**3.08**	3.04	3.10	3.19	3.19
T0341	2.48	2.46	2.48	2.46	2.48	2.46	2.48	2.48
T0380	2.51	2.52	**2.87**	**2.57**	2.26	2.21	2.26	2.23
T0332	2.66	1.94	**2.66**	**1.94**	2.66	2.44	**2.66**	**2.44**
T0298	2.70	2.58	**2.82**	**2.71**	2.81	2.72	2.82	2.73
T0368	2.87	2.85	**4.72**	**3.14**	3.50	2.91	**3.50**	**2.91**
T0313	3.05	3.10	**3.21**	**3.10**	3.12	3.12	3.12	3.15
T0375	3.28	3.26	**3.91**	**3.29**	4.34	4.26	4.34	4.34
T0373	3.56	3.58	**4.63**	**4.48**	3.61	3.61	3.61	3.61
T0385	3.64	3.60	3.64	3.60	3.64	3.60	3.64	3.64
T0349	3.84	3.79	3.84	3.79	3.84	3.54	**3.84**	**3.56**
T0384	3.84	3.86	**4.33**	**3.86**	4.37	4.61	4.61	4.61
T0330	3.99	3.94	4.02	3.94	4.02	4.12	4.02	4.01
T0317	4.18	4.22	4.77	4.78	5.51	5.49	**5.94**	**5.57**
T0369	4.25	3.90	**4.25**	**4.02**	4.32	4.28	4.32	4.33
T0297	4.50	4.50	**5.03**	**4.50**	5.03	5.02	5.03	5.03
T0331	4.68	4.56	**5.49**	**4.60**	4.86	4.86	4.89	4.86
T0327	4.90	4.87	**5.61**	**4.87**	4.90	4.83	**4.95**	**4.83**
T0348	5.82	5.29	**5.82**	**5.29**	5.82	5.68	5.82	5.93
T0371	6.15	6.16	**6.82**	**6.69**	6.15	6.15	**6.87**	**6.18**
T0383	6.26	6.21	6.31	6.22	6.01	5.90	6.03	6.01
T0312	6.63	6.55	**7.52**	**6.55**	8.30	8.14	**8.30**	**8.14**
T0370	6.81	6.52	**7.57**	**6.52**	6.81	6.51	**6.81**	**6.55**
T0350	7.22	7.22	**7.97**	**7.22**	5.04	5.04	5.04	5.04
Average	3.24	3.16	3.65	3.29	3.36	3.30	3.50	3.33

Table 2. Summary of the initial models and the refined CF models for 40 CASP7 targets.

The accuracies are evaluated using the GDT_TS scores between the model and the corresponding experimental structures. The best scoring CF model selected by the discriminatory function RAPDF is referred to “CF-R”; and the best initial model selected by RAPDF is referred to as “IN-R”. For mixing and matching between multiple initial models, the average GDT_TS score is improved from 0.733 to 0.744. The *P*-value from the Wilcoxon sign rank test between CF-R and IN-R is $8.941*10^{-4}$. Improvements above 0.01 between CF-R and INR are observed in 21 of 40 proteins (in bold font). For mixing and matching between two initial models, the average GDT_TS score is improved from 0.732 to 0.737. The *P*-value from the Wilcoxon sign rank test between CF-R and IN-R is 0.025. Improvements above 0.01 between CF-R and IN-R are observed in 9 of 40 proteins (in bold font). The evaluation by GDT_TS shows similar trend with that observed in the evaluation using CαRMSD calculation.

Target	Mixing and matching between multiple initial models				Mixing and matching between two initial models			
	Best initial model	Best CF model	Best scoring initial model	Best scoring CF Model	Best initial model	Best CF Model	Best scoring initial model	Best scoring CF model
T0346	0.990	0.996	**0.983**	**0.996**	0.990	0.996	0.990	0.996
T0290	0.984	0.994	**0.984**	**0.994**	0.984	0.993	**0.984**	**0.994**
T0345	0.961	0.964	**0.924**	**0.962**	0.961	0.964	0.961	0.962
T0315	0.894	0.895	0.894	0.893	0.901	0.903	0.897	0.900
T0305	0.921	0.915	0.914	0.915	0.914	0.915	0.914	0.915
T0302	0.835	0.830	**0.790**	**0.814**	0.835	0.814	0.790	0.803
T0308	0.908	0.904	0.901	0.904	0.901	0.915	0.901	0.909
T0291	0.919	0.917	0.900	0.893	0.916	0.906	0.910	0.899
T0324	0.802	0.797	**0.730**	**0.783**	0.802	0.802	0.802	0.802
T0328	0.819	0.823	0.819	0.820	0.819	0.818	**0.787**	**0.814**
T0340	0.908	0.908	0.906	0.906	0.908	0.911	0.906	0.908
T0288	0.843	0.860	**0.843**	**0.853**	0.843	0.843	0.843	0.843
T0303	0.775	0.779	**0.739**	**0.779**	0.775	0.779	0.775	0.775
T0295	0.766	0.772	0.761	0.770	0.758	0.764	**0.745**	**0.755**
T0359	0.823	0.820	0.823	0.804	0.809	0.819	**0.809**	**0.819**
T0362	0.779	0.786	**0.769**	**0.783**	0.764	0.766	0.764	0.760
T0335	0.714	0.714	**0.631**	**0.655**	0.655	0.661	0.631	0.631
T0341	0.720	0.724	0.720	0.724	0.720	0.724	0.720	0.720
T0380	0.768	0.757	0.753	0.757	0.773	0.780	0.773	0.780
T0332	0.832	0.849	**0.832**	**0.844**	0.832	0.833	0.832	0.833
T0298	0.707	0.717	**0.696**	**0.709**	0.707	0.717	**0.696**	**0.716**
T0368	0.723	0.721	**0.649**	**0.707**	0.671	0.683	**0.671**	**0.683**
T0313	0.793	0.787	0.786	0.787	0.716	0.717	0.716	0.716
T0375	0.640	0.640	**0.630**	**0.640**	0.612	0.620	0.598	0.598
T0373	0.635	0.631	**0.594**	**0.604**	0.651	0.651	0.651	0.651
T0385	0.780	0.797	**0.775**	**0.790**	0.775	0.790	0.775	0.775
T0349	0.662	0.652	0.662	0.649	0.662	0.659	0.662	0.659
T0384	0.699	0.702	**0.649**	**0.702**	0.646	0.638	0.638	0.638
T0330	0.597	0.603	**0.597**	**0.603**	0.597	0.597	0.597	0.594
T0317	0.799	0.799	0.793	0.791	0.771	0.785	**0.766**	**0.777**
T0369	0.619	0.622	0.619	0.622	0.541	0.529	0.541	0.527
T0297	0.687	0.690	0.687	0.681	0.687	0.691	0.687	0.687
T0331	0.692	0.710	**0.682**	**0.705**	0.664	0.647	0.642	0.642
T0327	0.647	0.660	**0.638**	**0.660**	0.644	0.660	**0.631**	**0.657**
T0348	0.578	0.582	0.578	0.582	0.578	0.578	0.578	0.578
T0371	0.614	0.618	0.614	0.604	0.606	0.608	0.606	0.601
T0383	0.528	0.556	**0.528**	**0.556**	0.564	0.564	**0.504**	**0.544**
T0312	0.511	0.470	0.511	0.470	0.475	0.452	0.448	0.452
T0370	0.596	0.600	**0.583**	**0.598**	0.596	0.600	0.596	0.587
T0350	0.433	0.386	0.433	0.386	0.532	0.532	0.532	0.532
Average	0.747	0.749	0.733	0.744	0.739	0.741	0.732	0.737
